# Myoxinol (Hydrolyzed *Hibiscus esculentus* Extract) in the Cure of Chronic Anal Fissure: Early Clinical and Functional Outcomes

**DOI:** 10.1155/2015/567920

**Published:** 2015-03-16

**Authors:** Adolfo Renzi, Antonio Brillantino, Giandomenico Di Sarno, Francesco D'Aniello, Stefania Ziccardi, Fiorella Paladino, Francesca Iacobellis

**Affiliations:** ^1^Pelvic Care Center, “Villa delle Querce” Hospital, Via Battistello Caracciolo 48, 80136 Naples, Italy; ^2^“A. Cardarelli” Hospital, Via Antonio Cardarelli 9, 80131 Naples, Italy; ^3^Second University of Naples, Piazza Miraglia 2, 80138 Naples, Italy; ^4^Radiology Department, Second University of Naples, Piazza Miraglia 2, 80138 Naples, Italy

## Abstract

*Objective.* This study was designed to evaluate the early results of the topical application of Hydrolyzed *Hibiscus esculentus* Extract 3% ointment (Myoxinol 3%), a novel local product with Botox-like activity, in the conservative treatment of chronic anal fissure (CAF). *Methods.* Among all patients with CAF observed during the study period, 31 subjects met the inclusion criteria and underwent medical therapy with Myoxinol 3% ointment every 12 hours for 6 weeks. Two patients were lost to follow-up. Clinical and manometric follow-up was carried out eight weeks after treatment. *Results.* At follow-up the success rate was 72.4% (21/29); median VAS score and mean anal resting pressure were significantly lower if compared with respective baseline data. The only one adverse effect of the topical application of Myoxinol 3% ointment was perianal itch, which was reported by 3,4% (1/29) of the patients available for the analysis. However, in this case this symptom did not cause interruption of the treatment. *Conclusions.* The topical application of Myoxinol 3% ointment in the cure of CAF shows encouraging early results. Further researches with a larger series and a longer follow-up are needed to confirm these data.

## 1. Introduction

Anal fissure is a common proctologic disease, characterized by an ulcer of the anoderm, usually located in the posterior midline and extending from the dentate line to the anal verge [1].

It represents the most common cause of severe anal pain and is associated with loss of working hours and reduced quality of life [2–5].

The etiology of chronic anal fissure (CAF) is still being debated and has not yet been completely clarified, even though increased anal tone and mucosal ischemia resulting from internal sphincter spasm represent possible pathogenetic mechanisms [6, 7].

Therefore, the principal therapeutic options are directed towards decreasing anal canal pressure by means of surgical procedures (lateral internal sphincterotomy), local application of vasodilators, or botulin toxin (BTX) injections [8–12].

Recently, a new local ointment for conservative treatment of CAF was introduced and contains, as its active principle, a 3% complex of oligopeptides (the so-called Myoxinol) obtained by biotransformation of native proteins of the seeds of* Hibiscus esculentus*.

These botanical oligopeptides showed in vitro demonstration of the “Botox-like” mechanism, obtaining genuine and reversible inhibition of muscle cell contraction, associated with anti-free-radical activity [13]. They already represent an alternative to Botox cosmetic injections in antiwrinkle treatment [14, 15].

However, the safety and efficacy of this local ointment in the treatment of CAF have never been evaluated and, therefore, the role of this new medical therapy in patients with CAF remains unclear.

This study was designed to evaluate the early results of local application of Hydrolyzed* Hibiscus esculentus* Extract 3% ointment (Myoxinol 3%) in the conservative therapy of CAF.

## 2. Methods

### 2.1. Study Population

Between January 2012 and December 2012 all patients with CAF (not healing in at least 6 weeks, with keratinous edges, fibers of the internal anal sphincter visible, and sentinel node or hypertrophied anal papillae present) were considered for enrolment in this prospective study. All the patients underwent clinical evaluation, physical examination, proctoscopy, anorectal manometry, and evaluation of anal pain intensity by means of a 10-point visual analogue scale (VAS) [16].

### 2.2. Inclusion/Exclusion Criteria

All the subjects with CAF, aged between 18 and 79 years and available to take part in this study, were included. Exclusion criteria included acute anal fissure, coexisting Crohn's disease or ulcerative colitis, TBC, HIV, pelvic radiotherapy or anorectal malignancies, anal incontinence, and previous medical and/or surgical treatment for CAF.

### 2.3. Study Design

After clinical assessment, all the patients who met the inclusion criteria underwent conservative treatment with Hydrolyzed* Hibiscus esculentus* Extract 3% ointment.

Briefly, the patients were instructed to apply 1 cm (approximately 220 mg) of the ointment with the tip of the index finger to a site just inside the anus at the junction of the perianal skin and the anal canal itself, twice daily at 12 hourly intervals, for 6 weeks. All the patients received a standardized advice to follow a high fiber diet, during the study period. No one standardized analgesic scheme was used. Pain was controlled by paracetamol plus codeine 500 mg tablets on patient's demand during the first week of treatment.

Two months after treatment, all the subjects underwent clinical assessment with physical examination, proctoscopy, evaluation of VAS score for anal pain, and anorectal manometry. All the patients showing treatment failure were offered the opportunity to receive both alternative conservative treatment with topical application of glyceryl trinitrate (GTN) 0.4% ointment and surgical therapy.

### 2.4. End Points

The primary and secondary end points of the study were, respectively, early success rate and incidence of adverse events. The treatment strategy was considered successful when the resolution of symptoms and the disappearance of fissure on physical examination were simultaneously obtained.

### 2.5. Anal Manometry

Anal manometry was performed using a silicone catheter with a diameter of 4.8 mm, provided with eight lumens and 4 radial openings located 5 cm from the tip, and perfused with twice distilled water through a low-compliance pump, at a constant rate of 0.75 mL per minute with 1.2 kg/cm^2^ flow (Menfis Biomedica, Bologna, Italy). The catheter was connected to a personal computer via pressure transducers.

The lubricated catheter was introduced manually into the rectum, with patients in the left lateral decubitus position and with flexed knees and hips.

The continuous pull-through technique was used with the catheter puller at a constant speed of 0.5 cm/s, for the evaluation of anal canal length (ACL), high pressure zone (HPZ), and mean resting pressure (MRP) [12]. The mean squeeze pressure (MSP) was recorded by means of the stationary pull-through technique.

Each investigation was repeated 3 times and the mean value was taken as the result.

The same manometric procedure was performed in a control group including 22 normal volunteers: 11 women and 11 men (mean age, 44 years; range, 18–64 years).

### 2.6. Statistical Method

The statistical analysis was carried out using the program InStat Graph-Pad Prism 5 (San Diego, California, USA).

Values are expressed as mean ± standard deviation (SD) or medians (range).

Continuous data were compared between each group using the Wilcoxon matched pairs test or the paired *t*-test, when indicated. Prevalence data were compared between groups using Fisher's exact test. A probability value of less than 0.05 was considered significant.

### 2.7. Ethics

The ethical committee of our institution approved the study protocol. All patients gave informed written consent.

## 3. Results

Among all the patients with CAF observed during the study period, 31 patients, who satisfied the selection criteria, accepted participation in this trial and were included in the study.

Out of these patients, 2 (6.4%) were lost to follow-up, whereas 29 (93.6%) (10 (34.4%) women and 19 (66.6%) men, median age 42 (range 19–61)) completed the entire follow-up and constituted the object of analysis.

### 3.1. Study Population

The most common symptom was anal pain, with a prevalence of 96.5% (28/29), whereas bleeding and constipation were reported, respectively, in 72.4% (21/29) and 51.7% (15/29) of cases. Two (6.8%) patients reported chronic diarrhea. At manometry, anal MRP in patients with CAF was significantly higher than in the control group (102.01 ± 9.3 versus 69.8 ± 12.4 mmHg (*P* < 0.0001; two sample *t*-tests)). Median VAS score was 6 (range 4–9).

### 3.2. Early Clinical and Functional Outcome

At eight-week follow-up the success rate was 72.4% (21/29).

Median VAS score and anal MRP were significantly lower if compared with respective baseline data (6 (range: 4–9) versus 3 (range: 0–7) (*P* < 0.0001; Wilcoxon matched pairs test) and 102.01 ± 9.3 mmHg versus 79.8 ± 13.4 (*P* < 0.0001; paired *t*-test)).

The only one adverse effect of local treatment with Myoxinol 3% ointment was perianal itch, which was reported by 3.4% (1/29) of the patients. However, this symptom was considered tolerable by the patient and did not cause interruption of the treatment. No case of anal incontinence to flatus or feces was observed.

The 8 patients with poor outcome refused prolonged medical therapy and, consequently, underwent surgery.

## 4. Discussion

Chronic anal fissure is a common proctologic disease with unclear pathophysiology and variable treatment strategies ranging from conservative therapy (chemical or pharmacological sphincterotomy) to surgery [17].

The goal of the treatment strategy is to decrease anal resting pressure in order to interrupt the vicious cycle of pain-spasm-ischemia involved in the arrest of fissure healing and self-maintenance of the disease [18].

At present, conservative medical therapy is considered the first-line treatment for CAF and can be based on topical application of vasodilators or BTX.

In the current study we reported preliminary results of topical application of a new local ointment (Myoxinol 3%) in patients with CAF.

This ointment is predominantly composed of low molecular weight oligopeptides obtained by biotransformation of native proteins from* Hibiscus esculentus*, a tropical plant native to Central Africa, India, Malaysia, and Philippines. Its seeds, widely used in traditional medicine, represent but a food source due to their high nutritional value.

This complex of oligopeptides was recently found to inhibit muscle contraction in vitro. This was demonstrated with an innovative in vitro model including a coculture of muscle cells with neurons that spontaneously displays rhythmic contractions. The inhibitory effect of Myoxinol was evaluated as a decrease in the frequency of contractions of the coculture matrix [13].

The evaluation of a muscle-relaxing mechanism was complemented by the detection of anti-free-radical activity in a battery of in vitro tests, covering primary free and secondary reactive oxygen species [14].

Based on the reported success rate of BTX injection [19–22], we hypothesized that the local application of a Botox-like and anti-free-radical activity ointment could be a safe and effective alternative in the conservative therapy of CAF.

This pilot study suggests that the topical use of Hydrolyzed* Hibiscus esculentus* Extract 3% ointment could determine, at short-term follow-up, a high percentage of success (72.4%) in the treatment of CAF, associated with a statistically significant reduction of anal MRP (79.8 versus 102.0 mmHg).

Our results are consistent with the antiradical and Botox-like properties of the adopted ointment and seem comparable, according to the literature [23], with results of topical calcium channel blockers and glyceryl trinitrate in the cure of chronic anal fissure.

Obviously, these preliminary data should be confirmed by further double-blind, placebo-controlled researches and randomized clinical trials with larger series.

In our study, the success rate at 2-month follow-up was 72.4%. We do not know if a prolonged treatment with the adopted ointment could increase the percentage of healed fissures or if the early good results could remain stable over time. However, these considerations go beyond the purpose of our preliminary study and other researches with longer follow-up should be encouraged to answer these questions.

Interestingly, in our series, the local application of the Hydrolyzed* Hibiscus esculentus* Extract 3% ointment was not associated with significant side effects. Indeed, only one patient reported perianal itch and no case of headache, hypotension, or anal incontinence was found. Moreover, the reported side effect was well tolerated and did not cause the interruption of treatment.

These findings, if confirmed, could encourage the use of Hydrolyzed* Hibiscus esculentus* Extract 3% ointment in clinical practice.

## 5. Conclusion

The local treatment of CAF with Hydrolyzed* Hibiscus esculentus* Extract 3% ointment (Myoxinol 3%) seems to show very encouraging early clinical results, with a high fissure healing rate and a statistically significant reduction of both anal spasm and pain. Further researches with larger series and longer follow-up are needed to confirm these data and to evaluate the mid- and long-term outcome of this novel conservative treatment for CAF.
